# Childbirth and Early Newborn Care Practices in 4 Provinces in China: A Comparison With WHO Recommendations

**DOI:** 10.9745/GHSP-D-18-00017

**Published:** 2018-10-03

**Authors:** Tao Xu, Qing Yue, Yan Wang, John Murray, Howard Sobel

**Affiliations:** a National Center for Women and Children's Health, Chinese Center for Disease Control and Prevention, Beijing, China.; b Reproductive, Maternal, Newborn, Child and Adolescent Health, Division of Noncommunicable Diseases and Health through Life-Course, World Health Organization, Regional Office for the Western Pacific, Manila, Philippines.

## Abstract

**Objectives:**

As a part of the process of implementing Early Essential Newborn Care (EENC) in China, which are evidence-based interventions recommended by the World Health Organization (WHO), we sought to understand whether current hospital policies are consistent with WHO-recommended standards and what factors influence their use. Data from the study will help inform policy changes needed to support the introduction of evidence-based childbirth and early newborn care practices effectively and to inform further scale up of EENC nationwide.

**Methods:**

Ten randomly selected hospitals in 4 early-introducing provinces participated in the study. We collected data from 20 simulated delivery scenario observations and focus group discussions and individual interviews with 10 hospital management staff. Policies, protocols, and guidelines related to childbirth and newborn care practice were also collected and reviewed at each hospital. Additionally, a survey was emailed to 15 childbirth and newborn experts from the 4 selected provinces and completed by 13. Data were compared with WHO EENC evidence-based standards to calculate the agreement rates. Barriers to introducing evidence-based guidelines were identified in focus groups and key informant interviews, then combined into common categories.

**Findings:**

Hospital policies were not consistent with WHO recommendations in 10 (59%) of the 17 delivery and early newborn care practices. Delayed cord clamping was recommended by 30% of hospital protocols and prolonged skin-to-skin contact by 13%, neither of which were observed in the delivery simulations. Kangaroo mother care (KMC) for stable preterm babies was required in only 17% of the hospitals; no preterm babies had KMC initiated, with all immediately separated from their mothers and admitted to neonatal intensive care units. Newborn resuscitation equipment was required to be placed within 2 meters of the delivery bed in 84% of hospital protocols, but was prepared in only 40% of cases. Immediate drying after birth was required in 48% of hospital protocols, but was initiated in only 20% of observed cases.

**Conclusions:**

Current childbirth and early newborn care policy and practice in China is not aligned with WHO recommendations for some major interventions. To make it easier and safer for hospital workers to practice EENC, expert working groups and national policies must be established to address inconsistencies and cultural beliefs and provide a strong, evidence-based set of guidelines for hospitals and health workers to follow.

## Abstract (precis)

In the 10 hospitals studied, we found that hospital policies, protocols, and interventions only partially align with WHO early newborn care recommendations, and that many hospitals still use outdated and non-medically sound practices.

## INTRODUCTION

With an estimated 16 million babies born annually, China has achieved remarkable progress reducing deaths among children under 5 over the past 2 decades. Between 1991 and 2015, the under-5 mortality rate of Chinese children declined by 80% from 79.2 to 10.7 per 1,000 live births and infant mortality declined from 50.2 to 8.1 per 1,000 live births.^1^ By 2015, the newborn mortality rate was 5.4 per 1,000 live births, which represented over half of all child deaths.^1^ Despite these reductions, the number of children under 5 dying each year remains close to 200,000, the majority of whom are born in remote rural areas without adequate care and support. In 2011, the World Health Organization (WHO) estimated that institutionalizing simple, low-cost interventions during childbirth and the early newborn period could prevent at least 22% of reported Chinese newborn deaths.^2^

Since the World Summit for Children in 1990, China has invested in strengthening policy and legislation for improving the child health system^3^^,^^4^ by enacting the *Law of the People's Republic of China on Maternal and Infant Health Care*^5^ and developing the *Measures for the Implementation of Law of the People's Republic of China on Maternal and Infant Health Care*^6^. Based on these 2 documents, the central government developed specific maternal and child health (MCH) action plans—the *National Program of Action Plan for Women Development in China (2011–2020)*^7^ and the *National Program of Action Plan for Children Development in China (2011–2020)*^7^—that identify 10-year objectives, main indicators to measure and meet, and strategies for improving women's and children's health, education, protection, and rights. While these documents covered a wide range of issues, quality early essential newborn care (EENC) was not mentioned.

Progress on improving the quality of care around delivery and in the early newborn period has been slower than other aspects of child health and recognized as an area that needs renewed attention.^8^ To that end, in 2013, China and 7 other countries, collaborated with the WHO Western Pacific Regional Office (WHO/WPRO) to develop and adopt the *Action Plan for Healthy Newborn Infants in the Western Pacific Region (2014–2020)*.^9^ This plan outlines an approach for implementing and scaling up a package of evidence-based EENC interventions recommended by WHO that have been demonstrated to reduce newborn mortality from the 3 most important causes: prematurity, birth asphyxia, and sepsis (Table 1). The EENC approach focuses on improving the quality, reach, and demand for facility-based maternal and newborn services using a systems-based approach to improve health worker practices.^10^ All 8 of the countries have since taken important steps in the areas of policy, planning, coordination, and program implementation. This has included local adaptation and endorsement of the *Early Essential Newborn Care Clinical Practice Pocket Guide*,^10^ the coaching/training of health facility staff on EENC, and institutionalization of approaches to improve the quality of practices in hospitals related to childbirth and the immediate newborn period.^11^

**TABLE 1.** Core EENC Interventions

Population	Intrapartum Care		Newborn Care
All mothers and newborn infants	The First Embrace	Labor monitoring (partograph)	Immediate drying Immediate skin-to-skin contact Appropriately timed clamping and cutting of the cord Exclusive breastfeeding Routine care (eye care, vitamin K, immunizations, weighing, and examinations)
At-risk mothers and newborn infants	Preterm and LBW infants	Preterm labor Elimination of unnecessary inductions and cesarean deliveries Antenatal steroids Antibiotics for preterm PROM	Kangaroo mother care Breastfeeding support Immediate treatment of suspected infection
	Sick newborn infants	Obstructed/prolonged labor Fetal distress Assisted delivery Cesarean delivery	Not breathing at birth Resuscitation Suspected sepsis Antibiotic treatment

Abbreviations: EENC, Early Essential Newborn Care; LBW, low birth weight; PROM, pre-labor rupture of membrane.

To improve quality of care during delivery and in the newborn period, 8 countries collaborated with WHO/WPRO to develop a regional action plan to implement and scale up EENC interventions.

In 2015, the National Health and Family Planning Commission (NHFPC) in China began prioritizing the introduction of EENC, beginning with improving quality of hospital care in 4 early-implementation provinces. Preliminary discussions with senior hospital staff found that hospital newborn health protocols often vary considerably within and across facilities. Prior to the implementation of EENC in China, we sought to first understand what protocols were being used in hospitals in the 4 early-implementation provinces, whether current policies were consistent with WHO-recommended standards, and what factors influence their use. The aim of these data was to inform the policy changes needed to support the introduction of evidence-based delivery and early newborn care practices effectively and to inform scale up to other regions of the country.

## METHODS

We conducted the study between December 2015 and April 2016 using observations of simulated deliveries, focus group discussions with the simulation participants, individual interviews with hospital management staff, a key informant survey mailed to Neonatal Resuscitation Program (NRP) instructors, and a desk review of hospital protocols.

### Study Sites

The study was conducted in Beijing, Shaanxi, Sichuan, and Inner Mongolia provinces. The NHFPC chose these 4 provinces because they are representative of provinces with different economic development statuses. In each province, 1 city and 1 county within this city were randomly selected. At each level—provincial, city, and county—1 hospital was randomly selected, for a total of 10 selected hospitals. Since Beijing is a municipality directly under the central government, only city- and county-level hospitals were selected.

### Simulated Delivery Observations, Focus Group Discussions, and Interviews

We conducted 2 simulated deliveries in each of the 10 hospitals selected, for a total of 20 simulated deliveries, and health care provider focus groups and interviews to assess current practices. We used scenario assessments instead of actual deliveries for 2 reasons: (1) we could not get permission from pregnant women to observe their actual deliveries, and (2) we believed that using simulated cases would be an easier and less stressful way for health care providers to demonstrate their routine practices.

**Figure:**
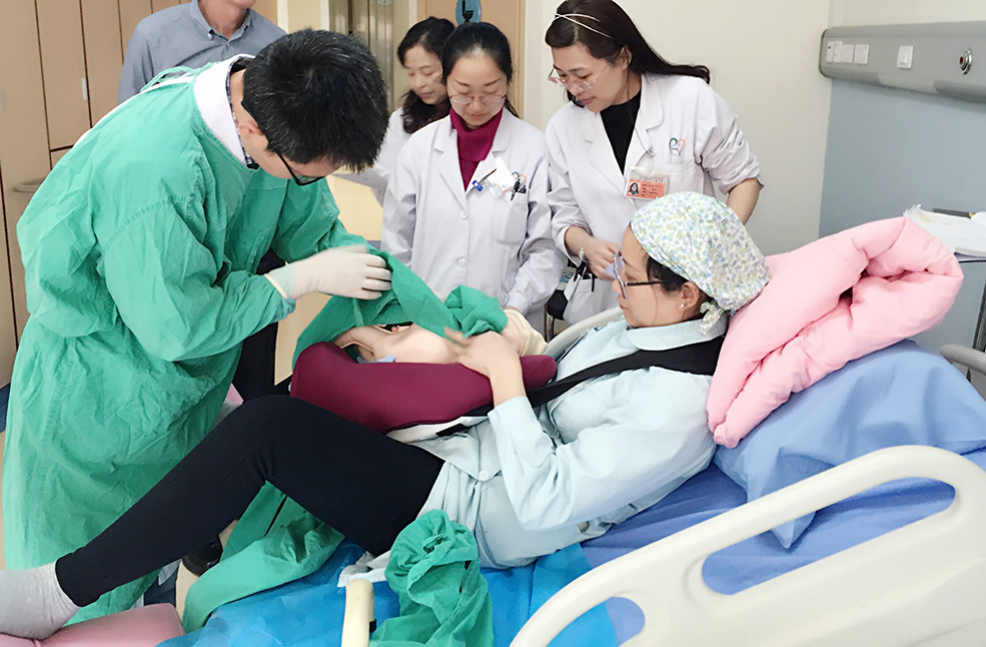
A simulated delivery scenario of a breathing baby was conducted in 1 hospital in Sichuan, China. © 2016 Tao Xu/National Center for Women and Children's Health, Chinese Center for Disease Control and Prevention

Simulated delivery scenarios, followed by focus group discussions, were used to assess current hospital and health worker practices without jeopardizing the health and safety of delivering mothers and their newborns.

Six staff members—2 midwives, 2 obstetricians, and 2 pediatricians—were selected randomly in each hospital to join a delivery simulation session and a focus group discussion. The sessions were conducted in a delivery room, or a room set up like a delivery room, and had 2 components. First, the facilitator asked a midwife and an obstetrician to demonstrate management of a routine normal delivery—dividing tasks according to the routine protocol—using a manikin infant, delivery kits, and other delivery room supplies. The facilitator observed and scored the tasks performed using a standard EENC skills review method and checklist, with 1 participant playing the role of the mother and the others conducting the delivery.^12^^,^^13^ Core practices reviewed were based on WHO recommendations and included in the EENC clinical coaching approach.^14^ The practice scenario was repeated for management of a non-breathing baby requiring resuscitation. Second, facilitators conducted a focus group discussion to identify steps that were inconsistent with WHO recommendations and probed why staff used these practices, what factors influenced the practices, what barriers impeded changing practices, and how compliance with standards was monitored.

In addition to the simulated delivery observations and the focus group discussions, 1 hospital management staff at each hospital was interviewed (N=10) about how the implementation of delivery and early newborn care policies was conducted, including how the hospital monitored adherence to hospital protocols and what was done if protocols were not followed. All observations, focus group discussions, and individual interviews were recorded for data management and analysis.

### Key Informant Mail Survey

A survey was emailed to 15 national NRP instructors in the 4 provinces to collect data on delivery and early newborn care policies, protocols, and guidelines. The group of NRP instructors included national and provincial health bureau managers, MCH program officers, and childbirth and newborn care specialists. A survey questionnaire was developed and distributed via email; respondents were asked to return the completed survey via email. The questionnaire asked respondents to compare WHO recommendations on key practices to current childbirth and newborn care practices, policies, and recommendations; describe how adherence to protocols within hospitals is monitored; and identify perceived barriers and challenges to implementing WHO-recommended guidelines.

### Desk Review of Hospital Protocols

At each of the hospitals, current policies, protocols, and guidelines related to childbirth and newborn care practice were also collected and reviewed. The documents reviewed included both practice guidelines (e.g., clinical procedures and standards) and management procedures (e.g., frequency of reporting of data, accreditation mechanisms). If protocols referred to primary sources, such as medical society recommendations, textbooks, or adapted international guidelines, these were also reviewed.

### Data Analysis

Data on the policies, protocols, and guidelines were extracted and entered into a Microsoft Office Excel 2013 file that allowed comparisons with WHO EENC evidence-based standards. Agreement rates for each practice or intervention were calculated by dividing the number of hospitals with protocols consistent with WHO recommendations by total number of hospitals. Each health care provider focus group discussion and interview was transcribed into a Microsoft Word 2013 file. Focus group and interview notes and transcripts were reviewed to summarize and compare the hospital protocols with WHO recommendations. Findings on accreditation mechanisms for current hospital protocols and perceived barriers to change current protocols were summarized across all hospitals and combined into common categories.

### Ethical Approval

Institutional ethics approval to conduct the study was obtained from the institutional review board at the National Center for Women and Children's Health at the Chinese Center for Disease Control and Prevention (NCWCH). Informed consent was obtained from all health care providers participating in observations and interviews.

## RESULTS

### Background Characteristics of Study Hospitals

Ten hospitals from 4 provinces were sampled. Site visits with desk review of policies, focus group discussions, individual interviews, and delivery observations were conducted at those hospitals. Thirteen of 15 mail survey questionnaires were returned. Respondents included 5 pediatricians, 3 obstetricians, 3 midwives, and 2 MCH program officers.

The number of annual deliveries and the number and type of staff available to provide delivery services varied greatly between hospitals (Table 2). In 9 of the hospitals, at least 1 midwife and 1 obstetrician are routinely present at each delivery, with the midwife practicing delivery under the supervision of the obstetrician. At 1 hospital, no midwives were available prior to 2015 and deliveries were conducted by obstetricians. Most hospitals had at least 1 nurse to help in the delivery room. Pediatricians were usually only present in delivery rooms for high-risk pregnancies or if they were called to help with emergency cases.

**TABLE 2.** Characteristics of Hospitals Included in the Study, China, December 2015

Province and Hospital	No. of Deliveries in 2014	No. of Obstetricians	No. of Pediatricians	No. of Midwives
**Beijing**				
Beijing MCH	17,250	68	15	65
Beijing University People's	2,343	16	17	12
**Shaanxi**				
Shaanxi Provincial MCH	13,338	111	81	55
Shangluo City MCH	1,845	18	12	9
Luonan County MCH	2,653	6	5	8
**Sichuan**				
Sichuan Provincial MCH	6,327	114	59	28
Liangshan City MCH	1,818	35	30	23
**Inner Mongolia**				
Inner Mongolia Provincial MCH	8,522	33	16	28
Wuhai City MCH	6,064	25	18	3
Nanhai County	394	6	4	4

Abbreviation: MCH, maternal and child health.

### Comparison of Hospital Protocols and Practices With WHO Recommendations

A review of hospital protocols—by self-report through the survey and onsite review by study staff—revealed that hospital protocols were consistent with WHO guidelines for 7 (41%) of the 17 delivery and early newborn care practices, including routine use of the partograph, use of corticosteroids for women of 24 to 34 weeks' gestation at risk of preterm birth, optimal timing technique for cord cutting, use of oxytocin for active management of the third stage of labor, routine administration of hepatitis B and bacillus Calmette-Guérin vaccines, and delayed bathing until at least 24 hours after birth (Table 3). Despite this, we also discovered that protocols for 10 practice areas were not consistent with WHO recommendations. None of the hospitals had policies on immediate and thorough drying after birth, delayed cord clamping, and dry-cord care. Less than 50% of hospitals had a policy on birth position and companion of choice during childbirth (38%), immediate and thorough drying after birth (48%), delayed cord clamping (30%), prolonged skin-to-skin contact (13%), delayed routine care until after the first breastfeeding (17%), and kangaroo mother care (KMC) for all newborns less than 2000 grams (17%).

**TABLE 3.** Number and Proportion of Hospitals With Delivery and Immediate Newborn Protocols and Practices Consistent With WHO Recommendations by Assessment Method, China, December 2015

Intervention	Protocol Self-Report via Mail Survey (n=13) No. (%)	Protocol Onsite Hospital Review (n=10) No. (%)	Observed Delivery Practice (n=10) No. (%)
Companion and position of choice for all deliveries	5 (39)	3 (30)	3 (30)
Maternal and fetal monitoring during labor including use of the partograph	13 (100)	10 (100)	10 (100)
Corticosteroids for women of 24 to 34 weeks' gestation who are at risk of preterm delivery	13 (100)	10 (100)	10 (100)
Bag and mask resuscitation kit available for every delivery, positioned within 2 meters of delivery bed	11 (85)	6 (60)	4 (40)
Drying started within 5 seconds after birth	7 (54)	4 (40)	2 (20)
Dried the baby thoroughly (wiped the eyes, face, head, front, back, arms, and legs)	0 (0)	0 (0)	0 (0)
No routine suctioning	0 (0)	0 (0)	0 (0)
Delayed cord clamping performed 1 to 3 minutes after birth, after cord pulsations have stopped	4 (31)	3 (30)	0 (0)
Clamp/tie placed at 2 cm, forceps at 5 cm from umbilical base	13 (100)	10 (100)	10 (100)
No placing substances on the cord stump	0 (0)	0 (0)	0 (0)
Skin-to-skin contact for a minimum of 90 minutes for newborns without complications	3 (23)	0 (0)	0 (0)
Intramuscular oxytocin given to mother within 1 minute	13 (100)	10 (100)	10 (100)
All routine newborn care (e.g., eye care, vitamin K, immunizations, and examinations) delayed until after a full breastfeeding	2 (15)	2 (20)	0 (0)
First dose of hepatitis B vaccine given within 24 hours of birth	13 (100)	10 (100)	10 (100)
Single dose of BCG vaccine given within 24 hours of birth	13 (100)	10 (100)	10 (100)
No bathing of the newborn until at least 24 hours after delivery	13 (100)	10 (100)	10 (100)
KMC for preterm babies weighing ≤2000 g at birth, including feeding with breast milk and monitoring for complications	3 (23)	1 (10)	0 (0)

Abbreviations: BCG, bacillus Calmette-Guérin; KMC, kangaroo mother care.

Hospital protocols and observed practices were consistent with only 41% of the 17 WHO-recommended delivery and early newborn care practices.

The practices of hospital staff who were observed in routine delivery scenarios were consistent with WHO recommendations in the same 7 practices areas noted for hospital protocols (Table 3). The other 10 practice areas were never or rarely practiced in observed delivery scenarios, regardless of the hospital protocol. Practices never conducted in practice scenarios included immediate and thorough drying after birth, immediate skin-to-skin contact of adequate duration, delayed cord clamping, absence of routine suction, and delaying routine tasks until after the first breastfeeding. Although delayed cord clamping was recommended by 30% of hospital protocols and prolonged skin-to-skin contact by 13%, neither were practiced in any case observations at the 10 hospitals, with hospital staff completing routine care (eye care, weight, and height) immediately after birth before skin-to-skin contact. Similarly, KMC for stable preterm babies was required in 17% hospitals; no preterm babies had KMC initiated, with all immediately separated from the mother and admitted to neonatal intensive care units. Newborn resuscitation equipment was required to be placed within 2 meters of the delivery bed in 84% of hospital protocols but was only prepared in 40% of cases. Immediate drying (within 5 seconds of birth) was required in 48% of hospital protocols but was initiated in only 20% of observed cases.

### Barriers to Improving Hospital Protocols for Delivery, Childbirth, and Early Newborn Care

Several potential barriers to introducing evidence-based guidelines were identified in focus group discussions with simulation participants and key informant interviews with NRP instructors. The results are summarized below.

Key barriers to improving hospital protocols included the lack of a standardized evidence-based set of clinical protocols and guidelines, supported by peer-reviewed literature and aligned with a system of evaluation.

#### Clinical Protocols and Guidelines

Each province has developed an MCH plan and the measures for administration of midwifery techniques guidelines. These provincial policies regulate the certification of delivery services, required preservice and in-service trainings and qualifications for providers, accreditation mechanisms and regulations, basic equipment and facility requirements, and content of services for the different levels of hospitals. However, none of these guidelines provide detailed clinical practice standards or protocols on immediate childbirth and early newborn care, leaving hospitals to look to other resources. For example, the *Guide for Prevention and Treatment of Postpartum Hemorrhage*^15^, developed by the Chinese Medical Society, provides detailed protocols for techniques such as cord clamping and the use of oxytocin, and an NRP guideline was developed for the NHFPC-led China NRP program. As a result, participants indicated that hospitals have to develop their own protocols based on the textbooks and guidelines available to them, which, in turn, has led to inconsistent protocols and practices across the different hospitals.

#### Systems of Accreditation and Legal Status of Protocols

While national- and provincial-level MCH service evaluation standards are currently available, they only focus on basic principles and requirements, not core evidence-based practices. Hospitals are evaluated every 3 or 4 years by provincial authorities. If the basic standards are not met, the hospital may be prohibited from providing MCH services the following year. If an individual staff member is found not to be practicing protocols, he or she may be disciplined by verbal criticism, deduction of wages, suspension of license, or a lawsuit, depending on the severity. Most hospital staff believe they should follow domestic textbook recommendations and Medical Society guidelines because these are officially sanctioned, and have legal status in case of medical disputes.

#### Understanding of the Evidence Base Supporting New Practices

Peer-reviewed journal articles used for WHO GRADE recommendations, for example, are felt to be important to explain key practice steps such as prolonged skin-to-skin contact, delayed cord clamping, non-use of suction except for non-breathing babies born with meconium staining and dry cord care. This is particularly important for explaining why new evidence-based recommendations are different from those used previously. For example, the previous duration for skin-to-skin contact recommended in baby-friendly hospital guidelines was 30 minutes, not 90 minutes. Similarly, many staff believed that applying disinfectants and covering the cord stump is important to prevent sepsis, and would like to see relevant data on this issue. In two hospitals, staff were concerned that mothers may not be able to safely hold babies in skin-to-skin contact and may drop them; in some cases there was concern that this position may be associated with an increase in the risk of asphyxia.

#### Cultural Beliefs and Practices

A number of cultural practices and beliefs held by families, particularly grandparents, prevent evidence-based practices from being applied including early separation of the newborn from the mother so the newborn can be shown to other family members, concerns that skin-to-skin contact with the mother is dangerous, a desire to bathe the newborn early, and beliefs that keeping the cord stump uncovered will allow “cool breezes” to pass through the cord stump into the newborn's body and cause illness.

#### Facility Support for New Practices

Maintaining skin-to-skin contact for 90 minutes, or until the first breastfeeding, usually requires the assistance of postnatal care staff who may not be familiar with the how and when to initiate each technique and for how long. Breastfeeding counseling, in particular, is essential for initiating the first feed. In some cases, staff members were concerned that beginning new practices would increase the workload for midwives or nursing staff and wanted clarification on how responsibilities would change.

## DISCUSSION

Our findings suggest that although China has no national EENC guidelines, many childbirth and newborn health care protocols and practices were evident in various documents at the hospital level. However, technical protocols related to childbirth and newborn health care were fragmented, outdated, and developed through a non-scientific guideline development process, and over half were not consistent with WHO guidelines. Because the EENC recommendations are new and not included in current protocols, facilities were not expected to adhere to them. To introduce EENC in China, implementers must recognize the need to identify necessary support for and changes in hospital policy, organization, accreditation mechanisms, and cultural beliefs.

Many of the technical childbirth and newborn health care protocols used by the hospitals studied were limited, outdated, and did not use a strict evidence-based guideline development process.

Since 2013, the Chinese NHFPC has been working with the United Nations Children's Fund (UNICEF) China to develop a newborn survival framework and service package, as the government response to the WHO and UNICEF Every Newborn Action Plan. The central and provincial health authorities have developed various policy documents that, for example, regulate the certification of delivery service, required preservice and in-service trainings and qualifications for providers, accreditation mechanisms and regulations, requirements for basic equipment and facilities, and a description of services for different levels of hospitals. In May 2018, the NHPFC issued the *Healthy Child Action Plan (2018–2020)*,^17^ and newborn health is one of the key areas that needs to be strengthened. These government documents do not, however, provide detailed technical protocols on childbirth and newborn health care interventions. Instead, hospitals have had to adopt and develop their own technical protocols based on textbooks, medical society guidelines, or experience learned from others. As a result, these hospital protocols were inconsistent in their scientific foundations and clinical procedures. In addition, although a description of childbirth and immediate newborn care was available in the hospital documents we examined, the technical procedures were fragmented and not presented or implemented in a systematic manner. To address policy and practice inconsistencies within and across hospitals in the country, a national guide on childbirth and early newborn care is needed.

In 1988, less than half of all women in China gave birth in hospital; within 20 years, hospital births became almost universal.^18^ This change is, in part, due to the government discouraging community midwifery and introducing a safe motherhood program that encourages hospital delivery in 2000.^19^ The Chinese NHFPC is now focusing on improving the quality of in-hospital maternal and child health care, especially the quality of care during and immediately after birth. WHO estimates that full implementation of EENC in the Western Pacific Region could prevent at least 50,000 newborn deaths each year.^9^ Central to EENC is the concept and practice of “The First Embrace,” a protected and prolonged skin-to-skin cuddle between mother and newborn, which allows proper warming, feeding, and cord care. The EENC protocol also includes the care of high-risk newborn infants, including preterm and low birth weight babies, and of sick newborn infants.^10^

Despite of these proven effective interventions, many inappropriate interventions are still practiced in hospitals that interfere with the baby's ability to adapt and feed well, such as unnecessary suctioning, immediate cord clamping, and delayed drying. These outdated practices increase the risk of delayed fetal-to-newborn circulatory adjustments, infection, breathing problems, hypothermia, anemia, acidosis, coagulation defects, brain hemorrhage, and trauma.^9^ Many newborns are distressed, hypothermic, and exposed to dangerous bacteria because they are separated from their mother.^10^ The first breastfeeding is often delayed because of an incorrect sequencing of actions taken immediately after birth.^7^ In our study, less than half of the hospital protocols we reviewed were consistent with WHO recommendations for procedures related to childbirth and immediate newborn care, such as immediate drying after birth, no routine suctioning, delaying cord clamping, skin-to-skin contact, no placing substances on the cord stump, and KMC for stable preterm babies. In addition, the abovementioned key practice areas were never or rarely practiced in observed delivery scenarios, regardless of the hospital protocol.

One recently published UNICEF reviews identified problems and bottlenecks in the health system to provide newborn care.^20^ Our research results support these findings and identified more specific barriers that health workers face introducing and practicing EENC in their facilities. First, there is no detailed national clinical practice guidelines for the management of routine delivery, childbirth, and immediate newborn care. As a result, policies and practices within and across hospitals were often not consistent. The knowledge on textbooks and experiences from other health workers were often outdated and harmful, and preservice and in-service trainings usually did not include sufficient instruction on quality EENC. As a result, many health workers were unaware that simple steps could protect newborn infants. Second, the protocols used must have legal validity for medical disputes and malpractice cases. The development and adoption of national evidence-based guidelines must therefore be initiated and approved by academic authorities or at the NHFPC level before being implemented in hospitals and incorporated into an effective accreditation mechanism. Third, the documents used for the evidence base should be made available to all stakeholders, including health workers and families of newborns. New practices need to be supported by an evidence base to secure widespread support from staff. In addition, the role of traditional and cultural beliefs must also need to be recognized, as they can influence how and why parents make certain decisions. Thus, capacity building activities should go beyond training and focus on coaching, which focus on methods for improving awareness of the importance of evidence-based practices. Last, insufficient coordination between obstetric and pediatric care complicates newborn care.^21^ Changes in facility support and the organization of work are required to support revised practices and to ensure that new practices are understood and adopted by all. For example, closer collaboration is needed between staff present at delivery who may currently divide or share tasks, particularly those tasks that obstetrics or midwifery staff may not traditionally feel is their responsibility, such as identifying whether a newborn is stable and able to be placed into immediate skin-to-skin contact and starting immediate newborn bag and mask resuscitation for non-breathing newborns.

The development and adoption of national evidence-based guidelines must be initiated and approved at the national level before being implemented in hospitals and incorporated into an effective accreditation mechanism.

Neonatal deaths in the Western Pacific Region declined slowly between 1990 and 2015.^9^ This was largely because of the widespread, outdated, and harmful health care provider practices.^11^ Through collaborative efforts with WHO and UNICEF, it is clear that countries in the region share many similar problems and barriers when scaling up EENC interventions. The results of this study may help countries working to ensure evidence-based policies and practices are used to improve their quality of skilled childbirth care.

## CONCLUSION

China has been working closely with various partners to prioritize newborn health by developing a national action plan and technical guideline that aligns with WHO recommendations. However, at the moment, hospital policies, protocols, and interventions only partially align with WHO recommendations. To make EENC easier and safer for hospital workers to practice, expert working groups and national policies must be established to address inconsistencies and cultural beliefs and provide a strong, evidence-based set of guidelines for hospitals and health workers to follow.

## Notes

**Peer Reviewed**

**Funding:** The research was funded by WHO/WPRO.
